# Physician-led team triage based on lean principles may be superior for efficiency and quality? A comparison of three emergency departments with different triage models

**DOI:** 10.1186/1757-7241-20-57

**Published:** 2012-08-20

**Authors:** Lena Burström, Martin Nordberg, Göran Örnung, Maaret Castrén, Tony Wiklund, Marie-Louise Engström, Mats Enlund

**Affiliations:** 1 Centre for Clinical Research, Uppsala University, Central Hospital, Västerås, Sweden; 2 Karolinska Institutet, Department of Clinical Science and Education, Södersjukhuset and Section of Emergency Medicine, Stockholm, Sweden; 3 Capio S:t Görans Hospital, Stockholm, Sweden

**Keywords:** Teamtriage, Lean principles, Emergency department, Quality indicators, Efficiency indicators, Mortality, Emergency physicians, Nurse

## Abstract

**Background:**

The management of emergency departments (EDs) principally involves maintaining effective patient flow and care. Different triage models are used today to achieve these two goals. The aim of this study was to compare the performance of different triage models used in three Swedish EDs. Using efficiency and quality indicators, we compared the following triage models: physician-led team triage, nurse first/emergency physician second, and nurse first/junior physician second.

**Methods:**

All data of patients arriving at the three EDs between 08:00- and 21:00 throughout 2008 were collected and merged into a database. The following efficiency indicators were measured: length of stay (LOS) including time to physician, time from physician to discharge, and 4-hour turnover rate. The following quality indicators were measured: rate of patients left before treatment was completed, unscheduled return within 24 and 72 hours, and mortality rate within 7 and 30 days.

**Results:**

Data from 147,579 patients were analysed. The median length of stay was 158 minutes for physician-led team triage, compared with 243 and 197 minutes for nurse/emergency physician and nurse/junior physician triage, respectively (p < 0.001). The rate of patients left before treatment was completed was 3.1% for physician-led team triage, 5.3% for nurse/emergency physician, and 9.6% for nurse/junior physician triage (p < 0.001). Further, the rates of unscheduled return within 24 hours were significantly lower for physician-led team triage, 1.0%, compared with 2.1%, and 2.5% for nurse/emergency physician, and nurse/junior physician, respectively (p < 0.001). The mortality rate within 7 days was 0.8% for physician-led team triage and 1.0% for the two other triage models (p < 0.001).

**Conclusions:**

Physician-led team triage seemed advantageous, both expressed as efficiency and quality indicators, compared with the two other models.

## Background

The principal management goals of emergency departments (ED) are safe and effective patient flow. However, studies from U.S. hospitals show that crowded EDs pose a risk to patient safety and delivery of quality of care [1-4]. Moreover, long waiting times negatively influence patient satisfaction [5-7], and may lead to patients leaving the ED before treatment is completed [8]. Unscheduled return for the same chief complaint may follow from a high proportion of patients leaving before treatment completed [9].

Many hospitals have their ED physicians mainly deployed in other clinics so their work in the ED is only sporadic. This arrangement may negatively impact on team-work, which in turn may lead to substandard patient care and reduced patient safety, particularly for severely ill patients [10]. Some EDs introduce emergency physicians with the aim of improving emergency care [11]. Different triage models have been introduced with the aim of providing safe care and adequate priorities. The three most common models used in Sweden are the Rapid Emergency Triage and Treatment System (RETTS), Adaptive Process Triage (ADAPT), and the Manchester Triage Scale (MTS). Assessment studies of such models, including descriptions of how they work, indicate that patient safety and waiting times may be improved when using a triage model [12-15]. However, the majority of models have been built around a main focus on critically ill patients. Adding lean principles to triage also implies a flow-optimized approach for all patients who come to the ED, regardless of the severity of their symptoms. Here, the patient will be met by a team that includes a physician [16]. Application of lean principles based on a flow process may shorten the time to first contact with a physician and lead to a shorter stay in the ED [17-19]. Lean thinking is a management strategy that is applicable to all organizations; it aims to improve streamlining processes, reduce cost, and improve the quality and timeliness of product and service delivery. The core of lean thinking involves determining the value of any given process by distinguishing value-added steps from non-value-added steps, to eliminate waste so that ultimately every step adds value to the process [20].

A recent Swedish systematic review of the evidence base for triage scales found that there is insufficient scientific evidence to determine whether there are differences between different triage models [21,22]. The aim of this multicentre study was to compare three Swedish EDs with different triage models in terms of efficiency and quality indicators.

## Methods

This retrospective study involved the EDs at three different hospitals in Sweden, two urban and one rural, with different patient receptions (Table 1). All patients included in the study were registered between 08:00 and 21:00 at each ED throughout 2008. This time was determined by the scheduling of triage between 08:00 and 21:00 in the participating ED that worked with lean principles and team triage. Because the proportions of attending children differed between the EDs, all attendants aged < 19 years were excluded to equate the EDs.

**Table 1:** Some characteristics of the three participating hospitals and their emergency departments (ED)

**Hospital**	**Catchment population (approx.)**	**Beds at hospital (approx.)**	**Attendants at ED**	**Age of visitors at ED**	**Working model at ED**
	**n**	**n**	**n**		
Capio S:t Görans Hospital	430000	310	64358	>15	Physician triage
Södersjukhuset	600000	450	91509	≥18/15	Nurse-physician triage
Central Hospital Västerås	251000	350	52271	All ages	Nurse triage

### Different working models

#### Physician-led team triage

**Figure 1:**
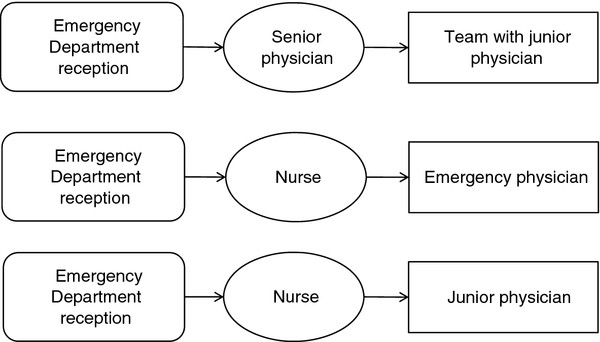
The principal organization of the three triage models studied.

A team triage system was developed at the urban hospital of Capio S:t Görans Hospital in 2007. The working model is a flow-oriented team triage led by a senior physician with a custom number of teams required for optimal patient flows. Each team, consisting of a junior physician and a nurse, has a detailed protocol for performing standardized work (Figure 1). All acute processes have been redesigned according to lean principles[20]. This working model is henceforth referred to as physician triage. The hospital does not use a standardized model of patient acuity assessment. The ED at this hospital is a trauma level II ED which provides acute medical, surgical and orthopaedic services. The ED serves adult individuals (minimum 15 years of age) (Table 1). The hospital has PCI fast track for patients with myocardial infarction and another fast track for patients with stroke. The ED staff train different kinds of students but no medical students.

#### Nurse/emergency physician triage

The second urban hospital, Södersjukhuset in Stockholm, has worked since 2008 with the nurse/emergency physician triage model, which has a registered nurse in front and an emergency physician in step two (Figure 1). The ED uses the ADAPT triage scale. This working model is henceforth referred to as nurse-physician triage. The general ED at this hospital is a trauma level II ED, and it serves adult individuals in acute internal medicine, cardiology, orthopaedics, (minimum 18 years of age) and surgery (minimum 15 years of age) (Table 1). The hospital has fast track PCI for patients with myocardial infarction and another fast track for patients with stroke. It also has a See-and-Treat area for patients with less acute conditions. Swedish and non-Swedish medical and non-medical students are in training at this ED.

#### Nurse/junior triage

The Central Hospital in Västerås works with the model of traditional single nurse triage at the ED. Following triage, the patient is examined by a junior physician (Figure 1). The ED uses a locally modified version of the MTS triage scale. This working model is henceforth referred to as nurse triage. The ED is a trauma level II ED, and it serves adults and children in five specialties: internal medicine, cardiology, surgery, orthopaedics, and gynaecology (Table 1). The hospital has fast track PCI for patients with myocardial infarction and another fast track for patients with stroke. Different kinds of medical and non-medical students are in training at the ED.

#### Staff hours

The total number of working hours for a specific ED staff member divided by the number of attendants was calculated to estimate any major discrepancies in staff density between the three spatially separated EDs (Table 2).

**Table 2:** Some characteristics of patients visiting the three emergency departments, mode of arrival and staff hours

	**Physician triage**		**Nurse-physician triage**		**Nurse triage**	
	**n**	**%**	**n**	**%**	**n**	**%**
**Attendants**						
Total number 08:00–21:00	49956	100	69661	100	41067	100
Male	21778	43.6	31712	48.1	16473	40.1
Female	25654	51.4	34182	49.1	17874	43.5
Children	1295	2.6	2352	3.4	6340	15.4
Missing data*	1229	2.5	1415	2.0	380	0.9
**In the study:**	47381	100	65880	100	34318	100
**Age groups**						
19-64	26411	55.7	39232	59.6	19685	57.4
65-79	9178	19.4	13586	20.6	7937	23.1
≥80	11792	24.9	13062	19.8	6696	19.5
**Attendants**						
Mode of arrival						
Ambulance	14546	29.9	20316	30.2	8383	24.1
Own means	34130	70.1	46993	69.8	26346	75.9
**Attendants**						
Education level						
< 12 years	25500	53.8	42588	64.6	22720	66.2
≥13 years	13462	28.4	16506	25.0	5575	16.2
Missing data*	4848	9.7	6089	9.2	2574	7.5
**Staff hours**						
08:00–21:00 Monday-Friday**	hours	hours/per head	hours	hours/per head	hours	hours/per head
Physicians	135.5	0.27	176.5	0.25	96.5	0.23
Nurses/Assistant nurses	240.5	0.48	221.5	0.31	274.0	0.66
**Staff hours**						
08:00–21:00 Saturday-Sunday**	hours	hours/per head	hours	hours/per head	hours	hours/per head
Physicians	102.5	0.20	110.5	0.15	74.0	0.18
Nurse/Assistant nurses	202.5	0.40	221.5	0.31	234.0	0.56

* The missing data resulted from attendants without a Swedish personal identity number, e.g. immigrants.

**Staff hours were calculated as the number of working hours for each staff category divided by the number of attendants.

### Ethics

The Regional Ethical Review board at Uppsala University, Uppsala, Sweden, approved the study (Approval number: 2009/414).

### Data collection

Data were collected from ED patient administrative computer systems used by personnel from the EDs to enter data and set time stamps. Data were then automatically collected in a database in each hospital, from which we have subsequently extracted the data. This procedure was identical for all three hospitals. Data were also extracted from the National Mortality Register. Data on patient education level were obtained from Statistics Sweden.

### Outcome definitions and measures

The following common definitions and measures are used in the current study [23].

*Length of stay* (LOS) = Time from registration to discharge.

*Time to physician* = Time from registration to being seen by a physician.

*Time from physician to discharge* = Time from being seen by a physician to discharge.

*4-hour turnover rate* = Proportion of patients spending less than 4 hours at the ED.

*24-hour unscheduled return* = Proportion of patients reattending the ED unplanned within 24 hours after the first visit for the same chief complaint.

*72-hour unscheduled return* = Proportion of patients reattending the ED unplanned within 72 hours after the first visit for the same chief complaint.

*Left before treatment completed* = Proportion of patients leaving the ED before treatment was completed.

*Mortality 7 days* = Proportion of patients dying within 7 days after the first visit to the ED.

*Mortality 30 days* = Proportion of patients dying within 30 days after the first visit to the ED.

### Statistical analyses

**Figure 2:**
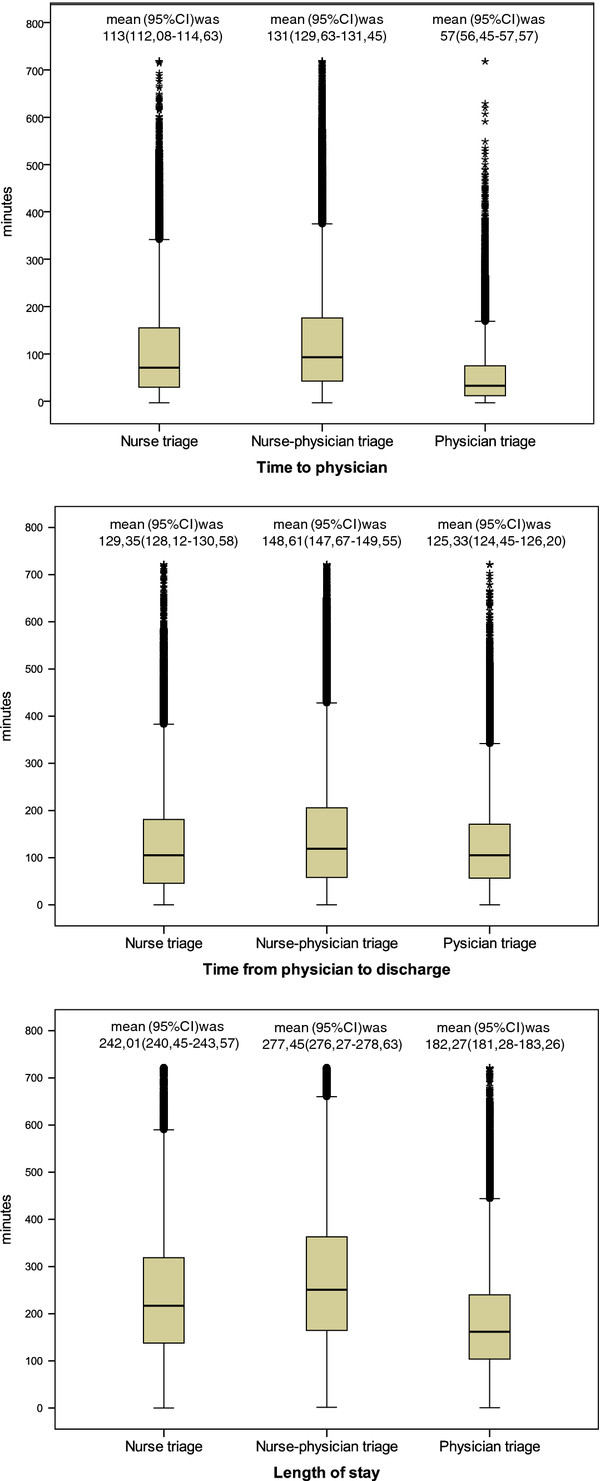
Length of stay and its time components at the three emergency departments.

Because of skewed distribution of the time variables, the Kruskal-Wallis test was used to investigate differences between hospitals (Figure 2). The Chi square test was used to analyse differences between the hospitals concerning the proportions of patients in different age groups, those spending less than 4 hours at the ED (Table 3), and for the analysis of quality indicators (Table 4). The Chi square test was also used to analyse differences between the hospitals in the proportions of patients in different age groups in relation to mortality within 7 and 30 days (Table 5). We used logistic regression to find predictors for 7 day mortality. Linear regression was used to find predictors for the time to being seen by a physician and for LOS. Data were analysed with the Statistical Package for the Social Sciences v 20 SPSS Inc., Chicago, Il, USA; a p-value < 0.05 was regarded as statistically significant.

**Table 3:** Numbers and proportions of patients spending less or more than four hours at the emergency department

	**Physician triage**		**Nurse-physician triage**		**Nurse triage**		**p-value**
	**n**	**%**	**n**	**%**	**n**	**%**	
Less than a 4 - hours stay at the ED	37043	76.1	32936	48.9	20511	59.0	<0.001
More than a 4 -hours stay at the ED	11595	23.8	34365	51.1	14218	40.9	<0.001
Missing data	66	0.13	14	0.02	31	0.08	
Total	48676	100	67309	100	34729	100	

**Table 4:** Quality indicators at the three emergency departments with different triage models

	**Physician triage**		**Nurse-physician triage**		**Nurse triage**		**Total**		**p-value**
	**n**	**%**	**n**	**%**	**n**	**%**	**n**	**%**	
Left before treatment completed	1557	3.1	3566	5.3	3344	9.6	8467	5.6	<0.001
Unscheduled return within 24 hours	649	1.0	1446	2.1	889	2.5	2984	2.0	<0.001
Unscheduled return within 72 hours	1195	2.4	2365	3.5	1304	3.7	4864	3.2	<0.001
Mortality within7 days after first visit	402	0.8	658	1.0	328	1.0	1388	0.9	<0.001
Mortality within 30 days after first visit	864	1.8	1281	1.9	685	2.0	2830	1.9	<0.001

**Table 5:** Mortality rates within 7 and 30 days in age groups for the three emergency departments (ED)

	**Age groups**	Physician triage*	Nurse-physician triage*	Nurse triage*	**p-value**
Mortality rate within 7 days after attending ED within age groups	19 - 64	0.10	0.22	0.18	
	65 - 79	0.85	1.03	1.21	<0.001
	> 79	2.52	3.32	2.94	
	Total	0.85	1.00	0.96	
Mortality rate within 30 days after attending ED within age groups	19 - 64	0.21	0.44	0.32	
	65 - 79	1.81	2.22	2.68	<0.001
	> 79	5.45	6.16	6.11	
	Total	1.82	1.94	2.00	

*number of deaths divided by the number of attendants per age group and ED.

## Results

Some characteristics of patients visiting the three EDs, their mode of arrival and staff hours are shown in Table 2*.* The proportion of elderly patients (>80 years of age) was highest at the ED using physician triage. Patients attending the ED using nurse-physician triage had the lowest education level, whereas those attending the ED using physician triage had the highest level. Arrival by ambulance was more common at the EDs with physician triage and nurse-physician triage. Staff hours were higher at the ED with nurse triage, especially during weekdays and for nurses/assistant nurses.

The six major chief complaints were the same at all EDs. The proportions between chief complaints varied to some extent between the EDs, with more of heart problems at the ED with physician triage and more of abdominal problems at the ED with nurse-physician triage.

### Missing data

The proportions of missing data on the different time components comprising LOS varied between the EDs from 0% to 9.4%. All missing data conceived the registration of intermediate time points, whereas data from the registration of patients coming into the ED and being discharged from the ED were complete. Thus, the quality markers were not affected by missing data.

### Efficiency outcome

The median LOS of 158 minutes was shortest at the ED with physician triage, whereas at the ED with nurse triage, it was 39 minutes longer, and at the ED with nurse-physician triage another 46 minutes longer (i.e., 85 minutes) (Figure 2). The time from physician to discharge constituted the main part of LOS with relatively small differences between the EDs. The major differences in LOS resulted from differences in time to physician. We also adjusted our model for waiting time for the confounders that included mode of arrival, sex, age, and originating hospital in linear regression analyses (not shown in tables). The only variable associated with waiting time was female sex (*r =* 0.027*,*p < 0.001). Furthermore, in the model with the ED with nurse-physician triage as reference, both the ED with nurse triage and physician triage had shorter waiting times (*r* = −0.067 and *r* = −0.321). The components in the regression models explained 9.2% (R^2^ = 0.092) of the differences in time to physician.

A similar pattern was discovered in an adjusted LOS regression model. In comparison with the EDs with nurse-physician triage and nurse triage, the ED with physician triage had shorter LOS (*r* = −0.113 and −0.307, respectively). Female sex (*r* = 0.046, p < 0.001) and age > 80 (*r* = 0.140, p < 0.001) were statistically related to LOS (*R*^*2*^ =0.100). Furthermore, a significantly higher proportion of patients at the ED with physician triage were treated within 4 hours compared with the other ED triage models (Table 3).

### Patient safety outcome

The ED with physician triage had the lowest proportion of patients leaving the ED before treatment was completed, and the ED with nurse triage had the highest proportion (Table 4). The proportions of unscheduled returns to the ED within 24 and 72 hours were significantly lower with physician triage compared with the other triage models (Table 4).

The rate of death within 7 days after the first visit to an ED was significantly higher at the EDs with nurse-physician triage and nurse triage compared with the physician triage (Table 4).

This was the case also for 30 days mortality and within different age groups (Table 5). Male sex (OR = 1.228, 95% CI = 1.104-1.366), age ≥60 (OR = 17.131, 95% CI = 13.715-21.397), and to be treated at the ED with nurse-physician triage (OR = 1.244, 95% CI = 1.098-1.410) were predictors of death within 7 days (Nagelkerke R^2^ = 0.093).

## Discussion

There were major discrepancies between the three EDs with their three different triage models in several outcome variables. Overall, the ED with physician-led triage based on lean principles seemed to be advantageous. These findings were not substantially changed even after adjustment for well-known confounding factors. However, a number of potential contributing factors must be discussed.

### Hospital characteristics

The proportion of attendants in relation to the number of inhabitants in the catchment areas was higher at the ED with nurse triage (Table 2). This rural ED is the only ED available for the inhabitants, whereas several EDs are available for the inhabitants in the major city, which gives patients who are not in need of ambulance transportation the opportunity to choose where they wish to go. The higher proportion of attendants in relation to the number of inhabitants could be a motive for a higher total number of staff at the ED with nurse triage.

### Patient characteristics: age, sex, education level, mode of arrival, and chief complaint

#### Age and sex

There was a significant difference in age and sex distribution between the EDs (Table 2). However, the associations of these two variables were weak in our multivariate models and were considered to be of no clinical importance.

#### Education level

The two EDs operating in a major city serve quite different populations, as illustrated in the different education levels between the two populations. Patients who attended the ED with physician triage, in a wealthy part of the city, were more educated than the patients attending nurse-physician triage, in a less wealthy part of the city. The well-known coupling between low education level and mortality might have to be taken into consideration here. The socio-economic gradient between the ED with physician triage and the ED with nurse triage was even greater, possibly explaining a higher demand for hospital resources at the hospital offering nurse triage [24]. However, at this ED there was a large group of children amongst the patients, all with poor or no education for obvious reasons. Thus, the average education level at this ED was “diluted”, which implies that the differences in outcome variables might not be explained by socio-economic differences. On the one hand, also outcomes, such as mortality, should also have been affected by the higher proportion of children, but this did not appear to be the case when we included children in the analyses (data not in Table).

#### Mode of arrival

How patients arrived at the ED was statistically significantly different between hospitals. There were higher proportions of patients arriving by ambulance at the EDs with physician triage and nurse-physician triage. According to the regression model, patients arriving by ambulance contributed to a shorter time from arrival to physician, although this association was weak. Clearly, this difference would relate to the fact that most of the patients arriving by ambulance would have been given a high priority, which would have reduced the time to physician.

#### Chief complaint

Although, we were unable to assess the level of urgency for our patients without having a common triage model in use in the participating EDs, the knowledge that each EDs is a trauma level II ED encouraged us to assume that the urgency of the patients did not differ much between the EDs. The six most common chief complaints were the same at all three hospitals with small differences in proportions between them. We assessed all chief complaints and further studied the top 28; the result was that the case mix was very much alike in all three hospitals. Although the proportions of patients arriving by ambulance differed statistically, it was just 5–6% less for nurse triage; as stated above, the degree of explanation for all components in the regression model was low.

A recent Swedish systematic review discussed the significance on death from hospitalization or 30 days after arrival at the ED [21]. The review stated that there is no uniform classification of chief complaints, and no study evaluated had enough scientific support for conclusions.

### Staff characteristics

There were more nurses and fewer physicians in service at the ED with nurse triage. The staffing power of physicians in the first line is often used in the description of the burden on an ED. The importance of having a physician in the first line was studied by Holroyd, who found that the proportion of patients who left before treatment was completed was lower compared with EDs with nurses in the first line [25]. It is worth noting that the ED with the best personnel resources (in total), namely nurse triage, performed worst in several quantitative and qualitative aspects. The presence of a high proportion of medical students may explain this to some extent, as may the fact that he ED with nurse-physician triage also had pedagogical tasks assigned to it. One may speculate that the ED triage model with a physician in the second line might contribute to the relatively poor results at the EDs with nurse triage and nurse-physician triage; the latter performed worst in the majority of performance indicators.

### Triage model and efficiency indicators

A political goal in many western societies is that a patient’s length of stay (LOS) at an ED should be no longer than four hours. In one benchmark study, a consensus group addressed the standardization of performance measures for emergency medicine. They reflected on LOS and came to the conclusion that a maximum six hour stay would be arbitrary and not useful. Moreover, they recommended the identification of outliers and consideration of why these were obstacles [26].

LOS was significantly longer at the ED with nurse-physician triage compared with the other two triage models (Figure 2). It clearly must take a longer time for a patient to meet two individuals in sequence instead of one. However that was also the procedure for nurse triage for which LOS was significantly shorter. Further explanations can be the relatively low figures for staff hours and the high total number of patients, which may render it difficult to create an efficient patient flow.

The significantly shorter LOS at the ED with physician triage was solely explained by time to physician, as the physician performed the triage. Moreover, the rest of the team was in place, able to perform their tasks according to the standardized protocols at the same time instead of acting in series. This ED triage model based on lean principles showed that a significantly higher proportion of patients was treated within four hours, which is in accordance with other studies (Table 3) [25]. In one study, in which LOS was described in relation to diagnosis, it appeared that there could be risks generated by placing too much emphasis on efficiency measures, such as LOS. The authors highlighted the importance of using multiple measures [27], a requirement that we have tried to meet.

### Triage model and quality indicator

Patients leaving before treatment is completed by a physician is a growing concern in overcrowded EDs, and it is a serious health issue that may delay care and result in adverse outcomes. Several factors may create crowding. We found a higher proportion of patients who left before treatment occurred with the EDs with nurse triage and nurse-physician triage. The lengthy waiting times to be seen by a physician with these triage models might contribute to, or even explain, the higher proportion of patients who left before treatment was completed, a finding that is in line with the results seen in an earlier study [8].

Several studies have used the rate of unscheduled return to the ED as a quality performance indicator [9]. However, there is no consensus on the length of time used to define unscheduled return, which ranges from 24 to 72 hours. In this study we used both time frames, and unscheduled return was significantly lower for both periods with physician triage (Table 5). The triage model based on lean principles with a senior physician in the first line might well explain this outcome.

Mortality within 7 days after the first visit to the EDs was significantly, but not remarkably lower with physician triage (0.8% vs 1.0% for the other two EDs) (Tables 4 and 5). The difference in 7-day mortality might be explained by the different triage models [28]. In the logistic regression, we found that male sex, high age, and treatment at nurse-physician triage were predictors for 7-day mortality, although with a low degree of explanation. The corresponding mortality within 30 days was 1.8% with physician triage, 1.9% at the ED with nurse-physician triage, and 2.0% at the ED with nurse triage.

### Strengths of the current study

The main strength of the current study was the large number of patients consulting the three EDs during the observation period of one year. The loss of data was low. From a statistical point of view, the study population may be regarded as the total population, rather than as a sample. Thus, the data may be considered as highly reliable.

### Limitations of the current study

Triage is only a part of patients’ treatments. Other matters and differences between the hospitals may affect survival. Different quality and efficiency indicators are used in different studies. Our choice of definitions was an attempt to adopt a “middle path”. Nevertheless, our definitions could be open to discussion. Data management could be a critical factor, especially as the data were gathered and merged from three different hospitals’ data systems. Finally, the original data were recorded by single individuals during busy working days, which might not be optimal for data gathering.

## Conclusion

This study indicated quite better results for the ED with physician- led team triage based on lean principles, as expressed in terms of both efficiency and quality aspects. The chosen triage model was most likely a major contributor to the result.

## Competing interests

The authors declare that they have no competing interests.

## Authors’ contributions

LB: study concept and design, acquisition of the data, analysis and interpretation of the data, drafting of the manuscript, obtained funding, administrative, technical and material support, and study supervision. MN: study concept and design, acquisition of the data, analysis and interpretation of the data, drafting of the manuscript, obtained funding. GÖ: study concept and design, acquisition of the data, analysis and interpretation of the data, drafting of the manuscript, obtained funding. MC: study concept and design, analysis and interpretation of the data, drafting of the manuscript, obtained funding. TW: data manager, administrative-technical- and material support. M-LE: study concept and design, analysis and interpretation of the data, drafting of the manuscript, obtained funding. ME: analysis and interpretation of the data, drafting of the manuscript, obtained funding. All authors read and approved the final manuscript.
